# The relationship between organizational communication and missed nursing care in oncology wards in Taiwan

**DOI:** 10.1002/nop2.976

**Published:** 2021-06-25

**Authors:** Shih‐Ping Pan, Chiou‐Fen Lin

**Affiliations:** ^1^ Department of Nursing Koo Foundation Sun Yat‐Sen Cancer Center Taipei Taiwan; ^2^ College of Nursing School of Gerontology Health Management Taipei Medical University Taipei Taiwan; ^3^ Department of Nursing Shuang Ho Hospital Taipei Medical University Taipei Taiwan

## Abstract

**Aim:**

Unfavourable communication increases missed nursing care. Oncology wards have more communication complexity than general wards; therefore, creating a positive communication environment is important for ensuring quality care. This study aimed to understand the relationship between organizational communication satisfaction and missed nursing care in Taiwan.

**Design:**

This cross‐sectional study was performed to measure organizational communication satisfaction and missed nursing care in six oncology wards at a stand‐alone cancer centre hospital in Taipei, Taiwan in December, 2018.

**Methods:**

The study collected data using the Communication Satisfaction Questionnaire and the MISSCARE survey. The data were analysed using descriptive statistics, *t* test, analysis of variance and Pearson product–moment correlation analysis in December 16, 2018.

**Results:**

A total of 111 questionnaires were collected, and the response rate was 92.5%. The study showed that nurses tended to miss nursing care when they were dissatisfied with the unit's manpower status organizational communication environment, horizontal and diagonal communication and informal communication.

## 1 INTRODUCTION

Medical error is attributed to a lack of attention by medical staff for items requiring attention. One type of medical error is missed nursing care, which is defined by Kalisch and Williams (2009) as a complete omission or delayed completion of certain routine care tasks by nurses. According to the Taiwan Patient Safety Reporting system, patient safety incidents are primarily caused by communication problems (Ministry of Health & Welfare, 2018), consistent with other studies on missed nursing care (Banerjee et al., 2016; Hessels et al., 2019; Kalisch et al., 2009; Prip et al., 2017). Communication is considered ideal when the thoughts and concepts received by the recipient are completely identical to those transmitted by the sender.

Establishing an effective communication plan in a medical organization is challenging, and patients with cancer are more prone to experiencing communication problems because of their complex treatment course (Epner & Baile, 2014; Henderson, Verrall et al., 2015). Studies on the severity and the primary reasons for missed nursing care and the correlation between communication problems and missed nursing care in the oncology units in Taiwan have not yet been explored. Hence, the current study was an important study that provided us with a better understanding of this topic.

## 2 BACKGROUND

A communication is considered ideal when the thoughts and concepts received by the recipient are completely identical to those transmitted by the sender.

Organizational communication is a complex interpersonal relationship (Robbins & Judge, 2014; Yen & Wei, 2011). Communication effectiveness can be measured by the perceived satisfaction of staff in a communication setting. (Robbins & Judge, 2014; Tsai, 2014; Yen & Wei, 2011). The Communication Satisfaction Questionnaire (CSQ) was developed by Downs and Hazen between 1973–1977 and is the most extensively used instrument for evaluating organizational communication effectiveness (Lee & Tsai, 1999; Tsai, 2014; Vermeir et al., 2017; Yen & Wei, 2011). The CSQ was translated into a Chinese version and validated as reliable in previous studies (Lee & Tsai, 1999; Tsai, 2014). Formal communication dimensions in the questionnaire include supervisory communication, communication climate, subordinate communication, communication with direct superior, overall organizational operation and horizontal and diagonal communication. For the informal communication, the questions focus on the perceived extent by which rumours in the company agree with the truth, perceived extent of the spread of rumours in the company, perceived attitude of company supervisors towards clique behaviours, extent of information one can obtain from rumours related to changes in the company, extent by which one can obtain from rumours related to the company's human resource arrangement; and extent by which one can obtain from rumours related to work performance evaluation results (Tsai, 2014). In the last 5 years, only one study focused on medical staff to examine the effects of communication satisfaction on individual and organizational fit; results showed that the communication satisfaction of hospital employees has significant positive effects on an organizational fit (Huang, 2015).

Kalisch et al. (2009) analysed the concept of missed nursing care according to their study results and developed a missed nursing care model. Thereafter, Kalisch and Williams (2009) designed an instrument to measure missed nursing care called the missed nursing care (MISSCARE) Survey. The MISSCARE Survey questionnaire was translated into different languages, and researchers successfully used this scale for related studies; however, the research results varied in terms of fields, settings or cultures (Chegini et al., 2020; Cho et al., 2015; Kalisch et al., 2011; Zeleníková et al., 2019) Studies concentrating on oncology wards include those of Friese et al. from the United States and Vryonides et al. from Europe. The common items for missed nursing care in oncology wards were turning and positioning of patients every 2 hr, attendance to cross‐functional/team meetings, oral care execution and ambulation thrice per day or as ordered. University graduate and age of 35 years and below were the primary attributes of nurses, and the greatest difference was observed in work experience. Most subject in the United States study had 5 years or less of work experience, whereas those in the European study had 5 years or more of work experience. Human resources, material resources and communication situation were the main causes of missed nursing care (Friese et al., 2013; Vryonides et al., 2018).

Most nursing managers believe that system structure and deficiencies in missed nursing care communication contribute to the development of missed nursing care (Dehghan‐Nayeri et al., 2018; Zeleníková et al., 2020). Medical institutions are complex organizations, and the staff must maintain patient safety by using effective communication (Vermeir et al., 2017). In 2006, the Joint Commission of Taiwan started promoting team resource management to a improve communication to reduce missed nursing care (Ministry of Health & Welfare, 2018), but the number of patient safety events reported persistently increased (Ministry of Health & Welfare, 2018). Studies on how communication affects missed nursing care are still missing. Therefore, based on the missed nursing care model that was developed by Kalisch, Landstrom, and Hinshaw (2009), the research questions that we aimed to answer were as follows:

1. What were the oncology nurses’ characteristics distributions?
2. What were the items of nursing care and the reasons for missed nursing care in oncology words? How were the items related with the reasons? What type of differences in characteristics could affect the items and the reasons?
3. How did the oncology nurses describe the organizational communication satisfaction? What type of differences in characteristics could affect their descriptions?
4. Did the organizational communication satisfaction relate to missed nursing care?

## 3 THE STUDY

### 3.1 Design

This study used a cross‐sectional study design. The research setting included all 6 oncology and haematology wards in a 200‐bed private specialty cancer hospital in Taipei, Taiwan. The study included nurses who were >20 years old and had worked in the oncology wards for >3 months. Nurses were excluded if they were on holiday, on probation or from other wards. All Registered nurses at the cancer hospital provided all types of care to people with cancer, and the average ratio of beds to nurses in the oncology wards ranged from 6–7 beds to one nurse. Of the nurses who participated in the study, 42 were aged ≤25 years, 49 were 26–34 years, 14 were 35–44 years and 1 was >45 years.

### 3.2 Methods

This study used a structured questionnaire. To determine the appropriate sample size for this study, a power analysis was performed using G*Power 3.1. The number of samples calculated by G*Power was 76. As all 120 nurses were present in these wards, this study distributed 120 questionnaires. The study's sample size was considered sufficient. The questionnaires were distributed to the participants in sealed envelopes during their ward meeting after explaining the nature and the scope of the study, together with an empty envelope to place their completed questionnaire, before placing it in the box that was left at the staff office for 2 weeks.

The questionnaire for this study included three major sections and the measurement, bias controls and quantitative variables were as follows:

1. General characteristics: This section comprised 15 items, namely, education level, rank, age, job title, working hours, number of years of nursing experience, shift type, overtime status, leave status, intention to resign, unit manpower, work load (admission/discharge service capacity), current work satisfaction status, work satisfaction and teamwork satisfaction.
2. MISSCARE Survey: Currently, the MISSCARE Survey does not have a Chinese version available. Thus, after the original author, Kalisch, granted copyright approval, the MISSCARE Survey was translated through the back‐translation method in May 2018. In August 2018, 100 nurses participated in a pilot study to analyse the validity and reliability of the Chinese version of the MISSCARE Survey. Three experts were invited to assess whether the cultural, conceptual and idiomatic equivalence of the original version was achieved. The results of the translated MISSCARE Survey questionnaire were as follows:
1. Validity analysis of the items of missed nursing care: The scoring system for this part was based on a five‐point Likert scale ranging from 1 (never missed) – 5 (always missed). The Kaiser–Meyer–Olkin (KMO) test and Bartlett's test of sphericity result for the items of missed nursing care was 0.954; the chi‐square value for Bartlett's test of sphericity was 3765.012, with a significance level of 0.400. The total reliability Cronbach's α was .982; therefore, with the total reliability of the questionnaire of >0.900, all questions in this section were retained.
2. Reliability and validity analysis results for reasons for missed nursing care by nurses: The scoring system for this part was based on a four‐point Likert scale ranging from 1 (significant related) – 4 (not related). The KMO was 0.865, the Chi‐square value for Bartlett's test of sphericity was 1027.002, and the significance level was <.001, which means significant. Therefore, this section was suitable for factor analysis. Next, principal component analysis was conducted, and the fixed factor value used for varimax rotation was 1. Finally, one factor was extracted, with an eigenvalue of 6.630. The cumulative explained variation was 39.001%; therefore, extracting one factor was reasonable. The factor load for questions 2 and 6 in this section was <0.400. However, these two questions are extremely important factors in other studies; therefore, they were not removed.
3. Furthermore, the total reliability Cronbach's α was .900. Therefore, all questions were retained.
4. Self‐perceived satisfaction on organizational communication in nurses: The Chinese version of CSQ was used after being approved by Professor Yuan‐Duen Lee. It includes seven dimensions, namely, upper management communication, communication climate, communication with subordinates, communication with direct supervisor, overall organizational operation, horizontal and diagonal communication and informal communication, with a total of 42 questions. For scoring, we used a seven‐point Likert scale (starting with 1 for “strongly disagree” and ending with 7 for “strongly agree”). Considering that the study subjects were frontline clinical nurses and the communication levels were limited because of actual job responsibilities, we only used communication climate, horizontal and diagonal communication and informal communication dimensions (18 questions) to focus on communication problems when nurses performed certain tasks. The validity and reliability test results for the 18 organizational communication questions are listed below:
1. Validity analysis of the organizational communication satisfaction: The KMO value was 0.909, the chi‐square value for Bartlett's test of sphericity was 2615.069, and the significance level was <.001, which means significant. Therefore, this section was suitable for factor analysis. Further, principal component analysis was conducted, and the fixed factor value used for varimax rotation was 1. Finally, three factors were extracted, with eigenvalues of 3.841, 4.956 and 3.841. The cumulative explained variation was 82.974%. Therefore, extracting three factors is reasonable. The factor load of all questions in this scale was >0.400; therefore, all questions were retained.
2. Reliability analysis of the organizational communication satisfaction: The total reliability score of the scale was 0.970. Given that the total reliability of the questionnaire was >0.900, the questionnaire has extremely good consistency. Therefore, all questions were retained.

### 3.3 Analysis

SPSS Version 19.0. Armonk, NY for Windows was used for analysis. Descriptive statistics that included percentages, mean and standard deviation (*SD*) were used to analyse the distribution of the participants’ characteristics, organizational communication satisfaction dimensions (including communication climate, horizontal and diagonal communication and informal communication dimensions) and item scores of the questionnaires. The scoring frequencies were determined to understand the distribution of the participants’ responses to 24 items of nursing care identified and evaluate the 11 reasons of missed nursing care.

Independent sample *t* test and one‐way analysis of variance were used to evaluate the differences between the participants’ characteristics and four organizational communication satisfaction dimensions or the items of nursing care (24 items divided into 4 categories: basic nursing‐related care procedures, nursing assessment, intervention measures and nursing plan) and evaluate the differences between the participants’ characteristics and the reasons of missed nursing care (11 reasons divided into 3 categories: human resources, material resources and communication status). If the *F* test of the single‐factor variance analysis was significant (α = .05), then the Scheffé method was used for post hoc comparison.

The Pearson product–moment correlation analysis was used to interpret the relationships among the nursing care items (a total of 24 items, including basic nursing‐related care procedures, nursing assessment, intervention measures and nursing plan), missed nursing care reasons (a total of 17 items, including human resources, material resources and communication status) and organizational communication satisfaction (including communication environment, parallel and diagonal communication and informal communication).

### Ethics

The participants were informed about the purpose of the research, the anticipated duration of the study and the procedures that would be used. They also were informed about any potential consequences of participating in the study, including potential risks, adverse effects or discomfort that may occur. After obtaining an Institutional Review Board approval from The Institutional Review Board /Ethics Committee of Koo Foundation Sun Yat‐Sen Cancer Center (Ethical approval number: 20180605A), the questionnaires were distributed to the participants. This study had an anonymous questionnaire, and the participants were allowed to withdraw at any time point.

## 4 RESULTS

A total of 111 valid questionnaires were received on 16 December 2018, corresponding to a response rate of 92.5%. Most of the respondents were university graduates, at N2 level (clinical ladder systems identify objective nursing competence classification as N1, N2, N3 and N4 in Taiwan), aged 26–34 years, worked on a 12‐hr day shift and had a total work experience of <2 years. Table 1 lists the full details of the participants’ demographic information.

**TABLE 1:** General characteristics of nursing staff

Item	*n*	Percentage
Education level		
Nursing school	12	11.3
University/2‐year technical programme/4‐year technical programme	94	88.7
Level		
N1	36	34.0
N2	61	57.5
N3	8	7.5
N4	1	0.9
Age		
≤25 years	42	39.6
26–34 years	49	46.2
35–44 years	14	13.2
>45 years	1	0.9
Job title		
Registered nurse	69	65.1
Registered nurse and team leader	37	34.9
Work shift		
Day shift	68	64.2
Graveyard shift	38	35.8
Number of years of nursing experience		
≤2 years	36	34.0
>2 years but ≤5 years	28	26.4
6–10 years	22	20.8
≥11 years	20	18.9
Shift type		
8 hr	2	1.9
12 hr	104	98.1
Overtime hours in the previous month		
0	24	22.6
1–12 hr	44	41.5
>12 hr	38	35.8
Expected time of resignation		
In half a year	7	6.6
In the following year	37	34.9
Not Considered	62	58.5
Unit manpower sufficiency status		
Always sufficient	2	1.9
Often sufficient	23	21.7
Occasionally sufficient	44	41.5
Rarely sufficient	16	15.1
Always insufficient	21	19.8
Mean number of patients cared for during the usual shift		
5–6	72	67.9
7–9	34	32.1
Mean number of newly admitted patients cared for during usual shift per day		
1–3	99	93.4
4–6	4	3.8
7–9	3	2.8
Mean number of discharging patients cared for during usual shift per day		
1–3	99	99
4–6	4	4
7–9	3	3
Degree of satisfaction on current work status		
Unsatisfied	7	6.6
Fair	38	35.8
Satisfied	53	50.0
Extremely satisfied	8	7.5
Degree of teamwork satisfaction		
Unsatisfied	4	3.8
Fair	23	21.7
Satisfied	65	61.3
Extremely satisfied	14	13.2

The top three items influencing high organizational communication satisfaction were the degree of work coordination between self and colleagues from one's assigned department or unit (mean ± *SD*, 5.52 ± 0.89), degree of communication for goal completion between self and colleagues from one's assigned department or unit (5.50 ± 0.92) and degree of streamlined commu ication between self and colleagues from one's assigned department or unit (5.42 ± 0.90) (Table 2).

**TABLE 2:** Analysis of organizational communication distribution among nursing staff

Question No.	Content of questions	Mean	*SD*	Rank
9	Degree of work coordination between me and colleagues from my department or unit	5.52	0.89	1
8	Degree of communication for goal completion between me and colleagues from my department or unit	5.50	0.92	2
7	Degree of streamline communication between me and colleagues from my department or unit	5.42	0.90	3

The top five items influencing missed nursing care were administration of stat medication orders in 15–30 min (4.21 ± 1.37), assessment of the effectiveness of medications after administration (4.18 ± 1.60), handwashing at suitable times (4.16 ± 1.59), completion of all nursing records as per schedule (4.09 ± 1.50) and medication administration in 30 min of the scheduled time (4.06 ± 1.34) (Table 3).

**TABLE 3:** Top five items influencing missed nursing care

Item	Mean	*SD*	Rank
STAT order medication administered in 15–30 min	4.21	1.37	1
Assess effectiveness of medications after they are administered	4.18	1.60	2
Hand washing at suitable times	4.16	1.59	3
Completion of all nursing records according to schedule	4.09	1.50	4
Medications administered in 30 min of scheduled time	4.06	1.34	5

The five main reasons for missed nursing care (Table 4) were the unexpected rise inpatient volume and/or acuity on the unit (3.73 ± 0.56), urgent patient situations (3.58 ± 0.65), insufficient number of nursing staff (3.44 ± 0.82), heavy admission and discharge activity (3.25 ± 0.83), and caregiver absence or unavailability (3.12 ± 0.85).

**TABLE 4:** Top five reasons for missed nursing care

Item	Mean	*SD*	Rank
Unexpected rise inpatient volume and/or acuity on the unit	3.73	0.56	1
Urgent patient situations (e.g. a patient's condition worsening)	3.58	0.65	2
Insufficient number of nursing staff	3.44	0.82	3
Heavy admission and discharge activity		0.83	4
Main caregiver (family member or nurse aide) not inpatient's room	3.12	0.85	5

The participants’ characteristics differences that would affect organizational communication include the nursing level (*F* = 5.47; *p* < .01), job title (*t* = 2.24; *p* < .01), unit manpower sufficiency status (*F* = 3.57; *p* < .01), degree of current job satisfaction (*F* = 10.28; *p* <.001) and degree of role satisfaction (*F* = 11.70; *p* < .001) and teamwork satisfaction (*F* = 9.0; *p* < .001) (Table 5).

**TABLE 5:** Correlations between the participants’ characteristics and organizational communication satisfaction

Characteristics	*n*	Communication climate	Horizontal and diagonal communication	Informal communication	Overall organizational communication satisfaction
		Mean ± *SD*	Mean ± *SD*	Mean ± *SD*	Mean ± *SD*
Level					
(1)N1	36	5.16 ± 1.25	5.63 ± 0.97	4.95 ± 1.14	5.25 ± 1.04
(2)N2	61	4.43 ± 0.93	5.08 ± 0.72	4.49 ± 0.84	4.66 ± 0.72
(3)N3/N4	9	4.80 ± 0.82	5.26 ± 0.68	4.39 ± 0.91	4.81 ± 0.71
*F*‐value		5.58**	5.34**	3.02	5.47**
*p*‐value		0.005	0.006	0.053	0.006
Scheffé method		1 > 2	1 > 2		1 > 2
Job title					
(1) RN	69	4.83 ± 1.13	5.41 ± 0.90	4.79 ± 1.01	5.01 ± 0.93
(2) RN and Team leader	37	4.47 ± 0.96	5.04 ± 0.68	4.35 ± 0.84	4.62 ± 0.71
*t*‐value		1.64	2.42*	2.28*	2.24*
*p*‐value		0.104	0.018	0.024	0.027
Unit manpower Sufficiency status Satisfaction					
(1) Always sufficient	2	5.67 ± 1.89	6.00 ± 1.41	5.58 ± 2.00	5.75 ± 1.77
(2) Often sufficient	23	5.42 ± 0.95	5.62 ± 0.78	5.05 ± 0.93	5.36 ± 0.83
(3) Occasionally sufficient	44	4.53 ± 0.98	5.19 ± 0.82	4.47 ± 0.92	4.73 ± 0.83
(4) Rarely sufficient	16	4.69 ± 1.15	5.30 ± 0.88	4.66 ± 1.08	4.88 ± 0.96
(5) Always insufficient	21	4.21 ± 0.97	5.03 ± 0.85	4.43 ± 0.87	4.56 ± 0.69
*F*‐value		4.86**	1.93	2.16	3.57**
*p*‐value		0.001	0.111	0.079	0.009
Scheffé method		2 > 3, 5			1, 2 > 5
Degree of current job satisfaction					
(1) Extremely satisfied	8	6.44 ± 0.63	6.21 ± 0.81	5.65 ± 1.24	6.10 ± 0.85
(2) Satisfied	53	4.84 ± 0.93	5.42 ± 0.79	4.75 ± 0.91	5.00 ± 0.79
(3) Fair	38	4.31 ± 0.78	4.90 ± 0.77	4.30 ± 0.86	4.50 ± 0.70
(4) Unsatisfied	7	3.93 ± 1.75	5.24 ± 0.68	4.48 ± 0.94	4.55 ± 1.01
*F*‐value		13.50***	7.32***	5.23**	10.28***
*p*‐value		<0.001	<0.001	0.002	<0.001
Scheffé method		1 > 2, 3	1, 2 > 3	1 > 3	1 > 2, 3, 4; 2 > 3
Degree of role satisfaction					
(1) Extremely satisfied	8	6.44 ± 0.63	6.21 ± 0.81	5.65 ± 1.24	6.10 ± 0.85
(2) Satisfied	60	4.79 ± 0.94	5.37 ± 0.78	4.73 ± 0.89	4.96 ± 0.79
(3) Fair	32	4.14 ± 0.54	4.84 ± 0.72	4.14 ± 0.69	4.37 ± 0.51
(4) Unsatisfied	6	4.67 ± 2.23	5.53 ± 0.92	5.00 ± 1.42	5.06 ± 1.43
*F*‐value		13.31***	7.66***	7.22***	11.70***
*p*‐value		<0.001	<0.001	<0.001	<0.001
Scheffé method for		1 > 2, 3, 4; 2 > 3	1 > 2 > 3	1, 2 > 3	1 > 2, 3,4; 2 > 3
Degree of teamwork satisfaction					
(1) Extremely satisfied	14	5.83 ± 1.03	5.89 ± 0.87	5.30 ± 1.16	5.67 ± 0.95
(2) Satisfied	65	4.73 ± 0.96	5.38 ± 0.77	4.66 ± 0.95	4.92 ± 0.80
(3) Fair	23	4.00 ± 0.75	4.71 ± 0.61	4.19 ± 0.58	4.30 ± 0.48
(4) Unsatisfied	4	4.54 ± 1.70	4.75 ± 1.25	4.58 ± 1.46	4.63 ± 1.42
*F*‐value		10.59***	8.18***	4.10**	9.01***
*p*‐value		<0.001	<0.001	0.009	<0.001
Scheffé method		1 > 2, 3, 4; 2 > 3	1, 2 > 3	1 > 3	1 > 2 > 3

Note

The descriptive data in each group are presented as mean ± *SD*.

* *p* < .05.

** *p* < .01.

*** *p* < .001.

Table 6 presents the correlations between the participants’ characteristics and the items of missed nursing care. The main reason identified that would most affect the items of missed nursing care was the intention to resign (*F* = 3.75; *p* < .05). Although the type of shift was statistically significant, given that the sample size difference was large (2 subjects for an 8‐hr shift and 104 for a 12‐hr shift), no significant correlation was observed in this study.

**TABLE 6:** Correlations between the participants’ characteristics and the items of nursing care

Characteristics	*n*	Nursing assessment	Intervention measures	Basic nursing‐related care procedures	Nursing plan	Overall items of nursing care
		Mean ± *SD*	Mean ± *SD*	Mean ± *SD*	Mean ± *SD*	Mean ± *SD*
Shift type						
(1) 8‐hr shift	2	4.78 ± 0.63	5.00 ± 0.94	4.08 ± 0.12	4.33 ± 0.47	4.55 ± 0.54
(2) 12‐hr shift	104	3.90 ± 1.27	4.01 ± 1.36	3.38 ± 0.77	3.76 ± 0.85	3.76 ± 0.98
*t*‐value		1.90	1.03	6.26**	0.95	2.00
*p*‐value		0.280	0.307	0.006	0.345	0.272
Intention to resign						
(1) In half a year	7	2.63 ± 0.23	2.74 ± 0.58	2.98 ± 0.52	2.95 ± 0.49	2.83 ± 0.23
(2) In the following year	37	4.03 ± 1.27	4.16 ± 1.40	3.43 ± 0.78	3.75 ± 0.67	3.84 ± 0.97
(3) Not Considered	62	3.99 ± 1.26	4.09 ± 1.33	3.42 ± 0.78	3.88 ± 0.92	3.85 ± 0.98
*F*‐value		4.09*	3.57*	1.13	3.98*	3.75*
*p*‐value		0.020	0.032	0.328	0.022	0.027
Scheffé method		2, 3 > 1	2, 3 > 1		3 > 1	2, 3 > 1

Note

The descriptive data in each group are presented as mean ± *SD*.

* *p* <.05.

** *p* <.01.

*** *p* <.001.

The human resource factors found to influence the reasons of missed nursing care were the nurses’ level (*F* = 3.23; *p* < .05), years of experience (*F* = 3.21; *p* < .05) and unit manpower sufficiency (*F* = 3.10; *p* < .05). The differences in level (*F* = 3.26; *p* < .05), job title (*t* = −2.27; *p* < .05) and intention to resign (*F* = 3.22; *p* < .05) were the communication status factors affecting the reasons of missed nursing care (Table 7).

**TABLE 7:** Correlations between the participants’ characteristics and the reasons of missed nursing care

Characteristics	*n*	Human resources	Material resources	Communication status	Overall reasons of missed nursing care
		Mean ± *SD*	Mean ± *SD*	Mean ± *SD*	Mean ± *SD*
Level					
(1) N1	36	2.97 ± 0.43	2.63 ± 0.39	2.06 ± 0.56	2.55 ± 0.34
(2) N2	61	3.17 ± 0.37	2.65 ± 0.42	2.38 ± 0.62	2.73 ± 0.28
(3) N3/N4	9	3.00 ± 0.32	2.48 ± 0.41	2.22 ± 0.57	2.57 ± 0.39
*F*‐value		3.23*	0.67	3.26*	4.12*
*p*‐value		0.044	0.514	0.043	0.019
Scheffé method		N.S.		2 > 1	2 > 1
Job title					
(1) RN	69	3.03 ± 0.41	2.61 ± 0.38	2.16 ± 0.60	2.60 ± 0.32
(2) RN and team leader	37	3.18 ± 0.37	2.67 ± 0.46	2.44 ± 0.58	2.76 ± 0.30
*t*‐value		−1.79	−0.70	−2.27*	−2.47*
*p*‐value		0.077	0.487	0.026	0.015
Years of nursing work experience					
(1) ≤2 years	36	2.93 ± 0.48	2.61 ± 0.43	2.07 ± 0.58	2.54 ± 0.35
(2) >2 years but ≤5 years	28	3.14 ± 0.30	2.67 ± 0.37	2.24 ± 0.68	2.68 ± 0.30
(3) 6–10 years	22	3.22 ± 0.28	2.55 ± 0.38	2.50 ± 0.53	2.75 ± 0.22
(4) ≥11 years	20	3.14 ± 0.40	2.70 ± 0.46	2.36 ± 0.57	2.73 ± 0.35
*F*‐value		3.21*	0.61	2.58	2.91*
*p*‐value		0.026	0.611	0.058	0.038
Scheffé method		N.S.			N.S.
Intention to resign					
(1) In half a year	7	2.94 ± 0.34	2.57 ± 0.46	1.73 ± 0.66	2.41 ± 0.36
(2) In the following year	37	3.09 ± 0.38	2.59 ± 0.31	2.35 ± 0.58	2.68 ± 0.28
(3) Not Considered	62	3.10 ± 0.42	2.66 ± 0.46	2.26 ± 0.60	2.67 ± 0.34
*F*‐value		0.47	0.33	3.22*	2.17
*p*‐value		0.624	0.717	0.044	0.119
Scheffé method				2 > 1	
Unit manpower sufficiency					
(1) Always sufficient	2	2.80 ± 0.28	2.50 ± 0.24	2.44 ± 1.26	2.58 ± 0.40
(2) Often sufficient	23	2.87 ± 0.52	2.64 ± 0.41	2.19 ± 0.65	2.57 ± 0.38
(3) Occasionally sufficient	44	3.11 ± 0.36	2.60 ± 0.38	2.25 ± 0.61	2.65 ± 0.33
(4) Rarely sufficient	16	3.18 ± 0.32	2.56 ± 0.32	2.22 ± 0.60	2.65 ± 0.27
(5) Always insufficient	21	3.23 ± 0.28	2.75 ± 0.53	2.36 ± 0.56	2.78 ± 0.26
*F*‐value		3.10*	0.65	0.28	1.24
*p*‐value		0.019	0.631	0.893	0.298
Scheffé method		N.S.			

Note

The descriptive data in each group are presented as mean ± *SD*.

* *p* < .05.

** *p* < .01.

*** *p* < .001.

Nursing assessment (*r* = .20, *p* < .05), intervention measures (*r* = .22, *p* < .05) and basic nursing‐related care procedures (*r* = .24, *p* < .05) were the top items of nursing care that were significantly correlated to the communication status. The responses indicate that communication status could lead to missed nursing care. The items of missed nursing care taken together were also significantly correlated to communication status (*r* = .21, *p* < .05) (Table 8).

**TABLE 8:** Correlations among the items of nursing care and reasons of missed nursing care

Items of nursing care	Reasons of missed nursing care			
	Human resources	Material resources	Communication status	Overall reasons of missed nursing care
Nursing assessment	0.05	0.01	0.20*	0.15
Intervention measures	0.08	0.01	0.22*	0.17
Basic nursing‐related care procedures	0.11	0.02	0.24*	0.21*
Nursing plan	0.01	0.05	0.10	0.09
Overall items of nursing care	0.07	0.02	0.21*	0.17

* *p* < .05.

** *p* < .01.

*** *p* < .001.

The analysis of correlation between the items of missed nursing care and the organizational communication satisfaction (Table 9) revealed that basic nursing‐related care procedures, and the items of nursing care overall were significantly correlated to the communication climate (*p* < .05, *r* = .20), horizontal and diagonal communication (*p* < .01, *r* = .28), informal communication (*p* < .05, *r* = .24) and overall organizational communication satisfaction (*p* < .01, *r* = .28). Human resource factors were also significantly correlated to the communication climate (*r* = −.22, *p* < .05), informal communication (*r* = −.20, *p* < .05), and satisfaction and overall organizational communication (*r* = −.19, *p* < .05).

**TABLE 9:** Correlations among the items of nursing care, reasons of missed nursing, and organizational communication satisfaction

Items of nursing care	Organizational communication satisfaction			
	Communication climate	Horizontal and diagonal communication	Informal communication	Overall organizational communication
Nursing assessment	0.00	0.10	0.07	0.06
Intervention measures	0.05	0.14	0.14	0.12
Basic nursing‐related care procedures	0.20*	0.28**	0.24*	0.27**
Nursing plan	0.00	0.07	0.04	0.03
Overall items of nursing care	0.06	0.15	0.13	0.12
Reasons of missed nursing care	–	–	–	–
Human resources	−0.22*	−0.09	−0.20*	−0.19*
Material resources	0.08	0.00	0.03	0.05
Communication status	0.02	0.02	0.02	0.02
Overall reasons of missed nursing care	−0.04	−0.03	−0.05	−0.05

* *p* < .05.

** *p* < .01.

*** *p* < .001.

## 5 DISCUSSION

Different results were obtained when the rank of items of missed nursing care in this study was compared with other studies. These differences may be attributed to staff characteristics. Most nurses who participated in this study were young and had <2 years of work experience. Some routine tasks do not require considerable attention or specialized knowledge to be completed (Papastavrou et al., 2014) and could be easily completed by less experienced nurses. Nursing is often fragmented and requires multitasking, and missed nursing care tended to occur because of the lack of continuity in tasks when nurses were inexperienced (Friese et al., 2015; Moreno‐Monsiváis et al., 2015).

In the current study, “insufficient number of staff” was the primary reason for missed nursing care. The institution in the study provided a nurse–patient ratio of 1:5 and 1:8 for the day and night shifts. The nurses still said that the number of staff was insufficient, particularly when an “unexpected rise inpatient volume and/or acuity on the unit,” “urgent patient situations” and “heavy admission and discharge activity” occurred. The study of Papastavrou et al. on oncology wards supports this phenomenon (Papastavrou et al., 2016; Villamin et al., 2019). Manpower management could be improved whether a flexible manpower dispatch system was well developed in the nursing information systems (NIS). NIS aims to simplify the staff's work processes. However, in this study, the nurses reported that NIS could not match their expectation. If NIS could have a reminder in a certain time to calculate a “busy state” based on patient care dynamics (number of delayed medication administration, discharge/admission volume and number of cases with abnormal vital signs), NIS could notify the unit manager to adjust the workload or to dispatch the manpower. Such functions could result to better on‐time coordination and decrease missed nursing care.

Nurse's characteristics, such as job level, job title, unit manpower, current job satisfaction status, role satisfaction and teamwork satisfaction were associated with organizational communication satisfaction, consistent with the results from previous studies (Kalisch & Lee., 2012; Prip et al., 2017; Vermeir et al., 2017; Villamin et al., 2019; Zeleníková et al., 2020). Most nurses in this study were from Generation Y and had a job rank of N2 (who has comparatively less knowledge and skills). The communication‐related characteristics of this generation of nurses are as follows: dislike hierarchy and authority figure, enjoy reform and teamwork, are immensely confident of their capabilities, can multitask and require task directions to be specific (Norouzinia et al., 2016; Sherman, 2015). In this study, Generation Y nurses with a lower job rank had higher organizational communication satisfaction and were more flexible in response to diverse work. Conversely, nursing team leaders were more experienced and had lower satisfaction. Factors, such as the years of work experience, education level, communication technique learning experience, situational anxiety and job title, are important because they affect the communication abilities of nurses (Prip et al., 2017; Vermeir et al., 2017). The team leaders in the present study were responsible for manpower allocation, and their communication satisfaction might be affected by the difficulties in responding to manpower requirements. The intention to resign is high in units with frequent occurrence of missed nursing care (Kalisch et al., ,,,^2011^, ^2013^), consistent with the present study. Our nurses believed that insufficient manpower affected their intention to resign.

Currently, the Joint Commission of Taiwan and hospital accreditations set the lowest number of required nurses. The institution that participated in this study has always had better manpower than others. However, manpower remains a factor affecting nursing care, indicating a gap in the definition of suitable manpower by the institution and nurses. Moreover, the present study showed that when missed nursing care showed a higher correlation with manpower resources, satisfaction in communication climate and informal communication would decrease. Communication affects organizational behaviour; when communication satisfaction is high, the probability of unexpected behaviour will decrease (Chan & Lai, 2017; Wagner et al., 2015).

To avoid missed nursing care, the nursing management needs to examine whether the organizational ethical climate benefits the management of missed nursing care. An ethical climate is important in an organizational climate and is considered to be the common belief and value of a company. When the organizational ethical climate is caring oriented, nurses are less probably to rely on individual values to determine nursing care priority. They would follow the consensus of practice and decrease missed nursing care (Vryonides et al., 2018).

## 6 LIMITATIONS

This study had two major limitations that could be addressed in the future research. First, the selection of nurses from a single region in a cancer centre in Taiwan did not allow the extrapolation of the results to the entire country or other types of care units. Second, cross‐sectional design and self‐reported data may be sources of potential biases.

## 7 CONCLUSION

This study showed that the nurses tended to miss a part of nursing care when the communication status reason exists. When the nurses were performing basic nursing‐related care procedures, these procedures could be missed whether they were not satisfied with the unit manpower status and the organizational communication climate, horizontal and diagonal communication and informal communication. The results can be used to supplement future studies for missed nursing care in clinical practice.

This study recommends using a scenario simulation training to enhance the nurses’ communication skills and creating an on‐the‐job education module according to the needs of different levels and ages. Moreover, the missing nursing conceptual framework points out that the internal processes of the nurses will affect the implementation of the final nursing priorities and there would be omissions; therefore, this study suggests that managers should pay attention to the impact of organizational ethics on missed nursing care and communication problems. The MISSCARE Survey Chinese version is valid and reliable. Hence, this version was used to preliminarily examine the relationship between missed nursing care and organizational communication satisfaction in a cancer centre in Taiwan.

## CONFLICT OF INTEREST

There is no author has any conflict of interest to disclose.

## ETHICAL APPROVAL

The study is approved by The Institutional Review Board /Ethics Committee of Koo Foundation Sun Yat‐Sen Cancer Center (Ethical approval number: 20180605A). All procedures in this study are carried out in accordance with the principles of Declaration of Helsinki. Informed consent for participation was not required. Since this study has an anonymous questionnaire, and the participants can withdraw at any time.

## Data Availability

The data that support the findings of this study are available from the corresponding author upon reasonable request.
